# Supplementary material for: Recapitulation of selective nuclear import and export with a perfectly repeated 12mer GLFG peptide
Source: Nat Commun. 2021 Jun 30;12:4047. doi: 10.1038/s41467-021-24292-5 (PMC8245513; doi:10.1038/s41467-021-24292-5)
Supplement: Supplementary file 1 — Supplementary Information [file 41467_2021_24292_MOESM1_ESM.pdf]

Supplementary Information:

Recapitulation of selective nuclear import and export with a perfectly repeated 12mer GLFG peptide

Sheung Chun Ng, Thomas Güttler, and Dirk Görlich*

Department of Cellular Logistics, Max Planck Institute for Biophysical Chemistry, Göttingen, Germany

*To whom correspondence should be addressed. E-mail: goerlich@mpibpc.mpg.de

Contents:

Supplementary Figures 1-9 with legends

Supplementary Tables 1-4

Supplementary Note 1: Complete amino acid sequences of engineered FG domain variants and reference wild-type FG domains

Supplementary References

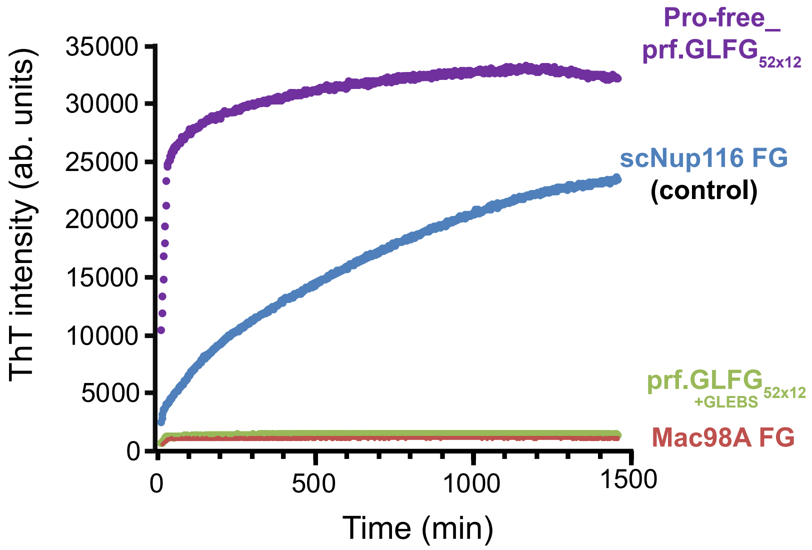

Supplementary Figure 1: The proline-free, perfectly repeated GLFG12mer phase rapidly transforms into an amyloid

300µg of indicated FG repeat domains were dissolved at 65µg/µl in guanidinium hydrochloride and rapidly diluted with 50x volumes of buffer (50mM Tris/HCl pH 7.5, 150mM NaCl, 5mM DTT) to initiate phase separation. 2 mins later, 20µM Thioflavin-T (ThT) was added, and fluorescent signals were recorded at 2-min intervals (excitation: 446 nm; detection: 477-487 nm). ThT fluorescence reports amyloid cross β-sheets. Amyloids typically form on timescales of hours[1,2], as exemplified by the Nup116 FG domain. The Pro-free_prf.GLFG$_{52x12}$ phase, however, transformed within minutes into an amyloid.

Note that an 'aged' (≈17 hours) Nup116 FG phase stained rapidly upon ThT addition, indicating that amyloid structures are reported but not induced by the dye.

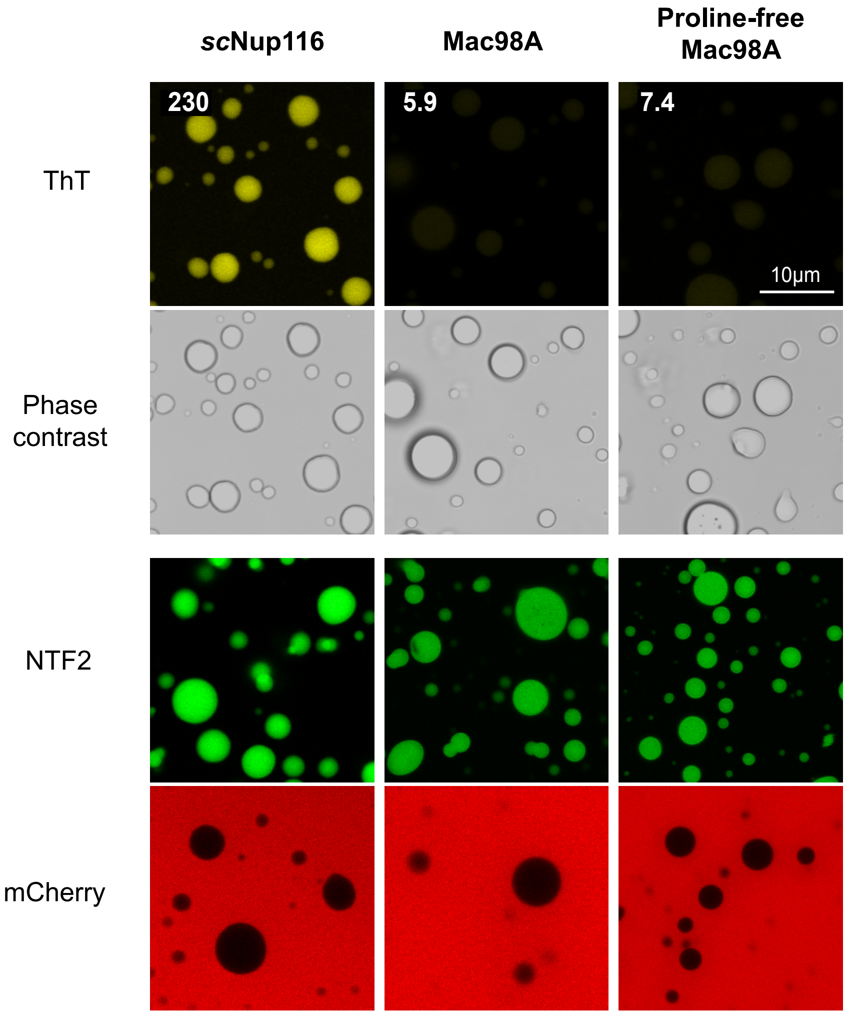

Supplementary Figure 2: ThT staining is not enhanced by proline replacement in the non-perfectly-repetitive wildtype MacNup98A FG domain (Mac98A)
Proline-free Mac98A was derived from Mac98A by substituting all 31 prolines in the N- and C-terminal FG repeats by other amino acids. The GLEBS domain was not changed. Proline-free Mac98A self-assembled into an FG phase that stained only weakly with ThT while showing transport selectivity and morphology like the wildtype Mac98A. ThT-staining intensities (as FG in:out) are indicated by the numbers. Sequences are listed below (Supplementary Note 1).

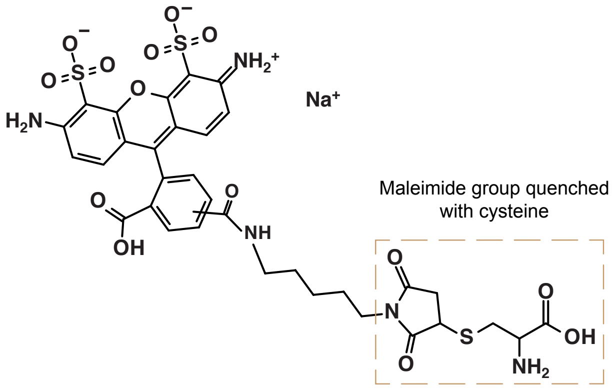

Mac98A FG inside:outside= 6.3

Alexa488 (Alexa Fluor 488) maleimide quenched with cysteine

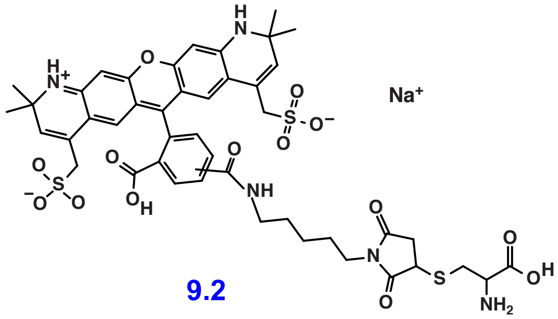

9.2

Alexa568 (Alexa Fluor 568) maleimide quenched with cysteine

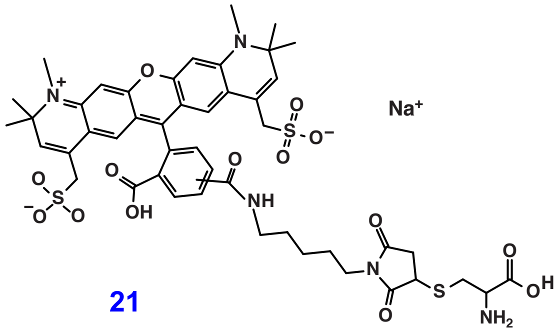

21

Alexa594 (Alexa Fluor 594) maleimide quenched with cysteine

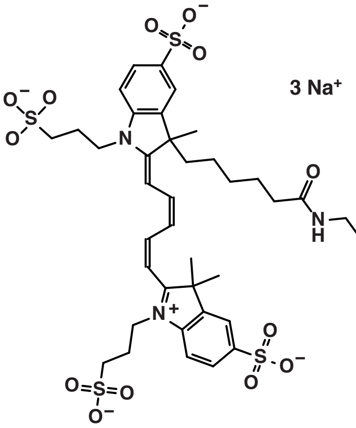

8.5

Alexa647 (Alexa Fluor 647) maleimide quenched with cysteine

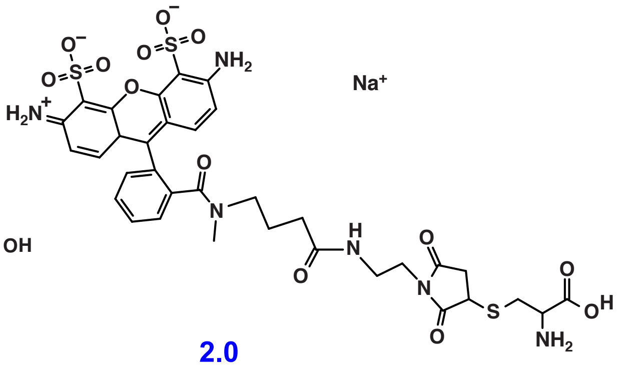

2.0

Atto488 maleimide quenched with cysteine

Supplementary Figure 3: Chemical structures of fluorophores tested in this study (according to the suppliers)

The structures of Abberior STAR635P, DyLight405 and DyLight488 maleimides were not included as they were not disclosed by the suppliers. Alexa488/ Alexa568/ Alexa594/ Atto565: mixtures of 5- and 6-isomers. Blue numbers: ratios of fluorescence inside:outside the Mac98A FG phase, after incubating FG particles with 2-9µM of the fluorophore (see the main text).

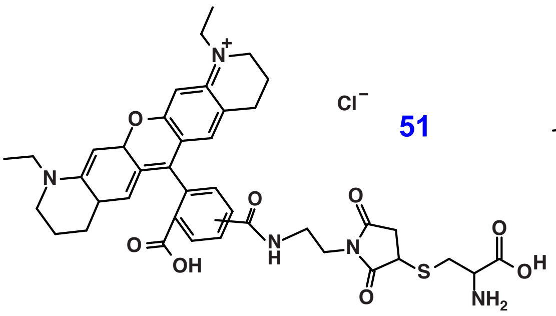

51

Atto565 maleimide
quenched with cysteine

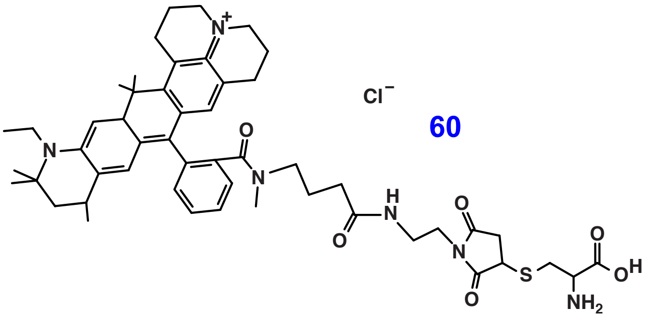

60

Atto647N maleimide
quenched with cysteine

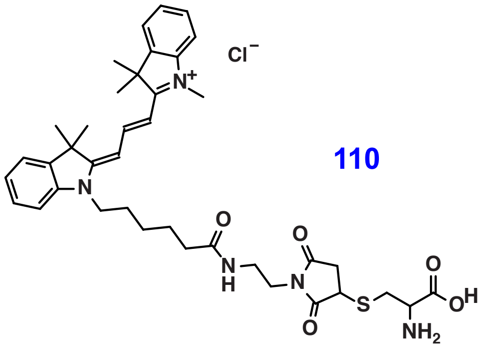

110

Cy3 maleimide quenched
with cysteine

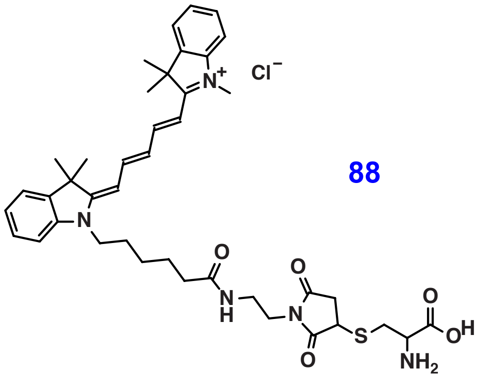

88

Cy5 maleimide quenched
with cysteine

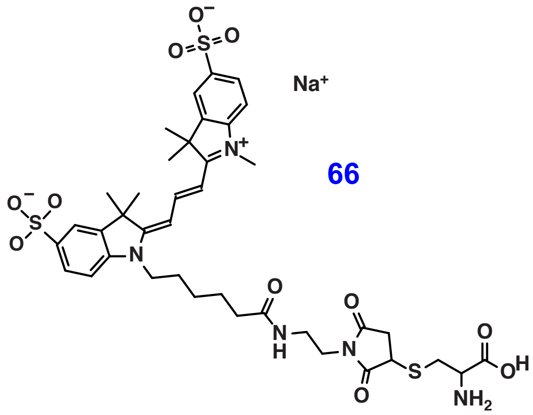

66

Sulfo-Cy3 maleimide
quenched with cysteine

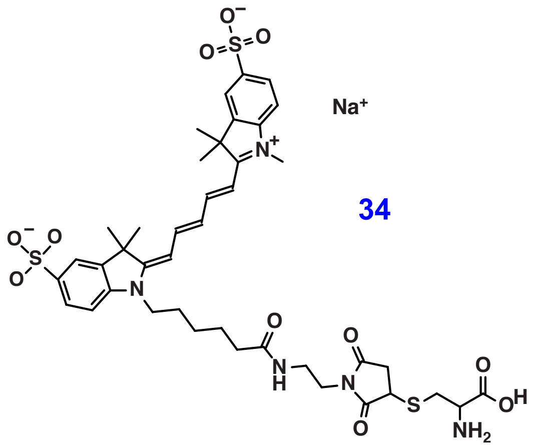

34

Sulfo-Cy5 maleimide
quenched with cysteine

Supplementary Figure 3: Chemical structures of fluorophores tested in this study (continued)

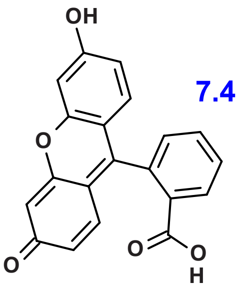

7.4

Fluorescein

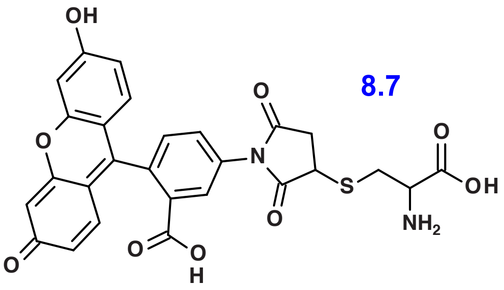

8.7

Fluorescein maleimide quenched with cysteine

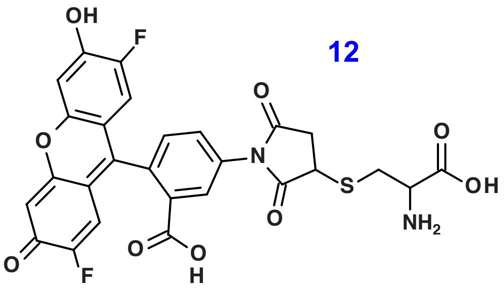

12

Oregon Green 488 maleimide quenched with cysteine

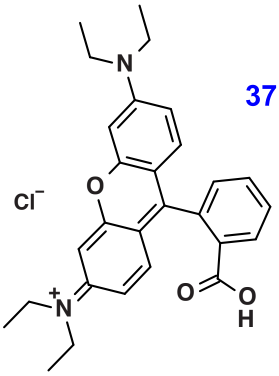

37

Rhodamine B

1700

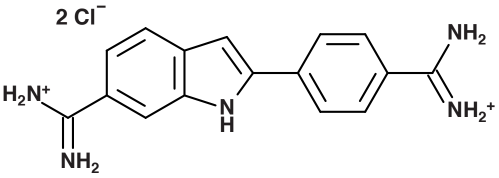

DAPI (4’, 6-diamidino-2-phenyl-indole)

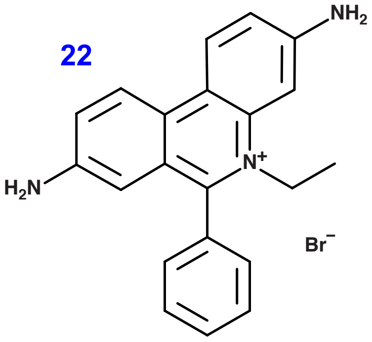

22

EtBr (Ethidium bromide)

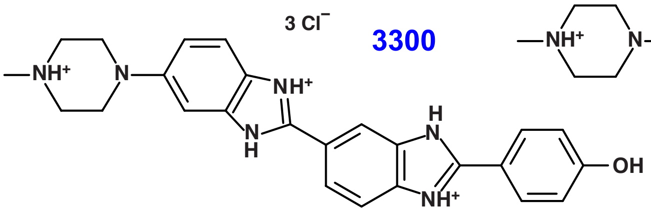

3300

Hoechst 33258

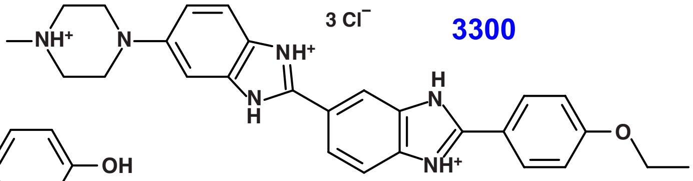

3300

Hoechst 33342

Supplementary Figure 3: Chemical structures of fluorophores tested in this study (continued)

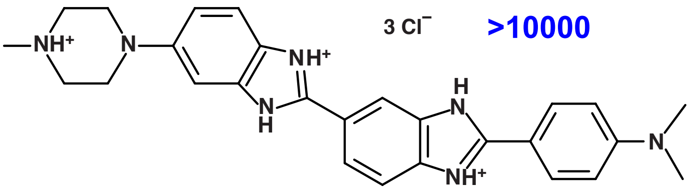

Hoechst 34580

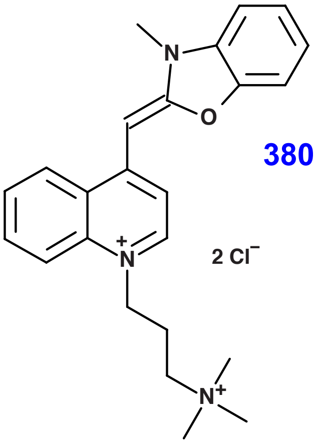

Oxazole Yellow

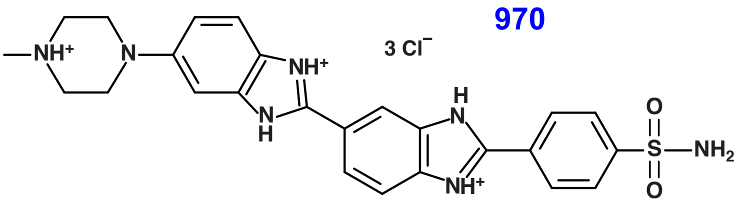

Nuclear Yellow

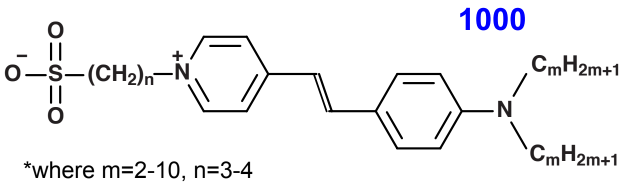

*where m=2-10, n=3-4

SYPRO orange

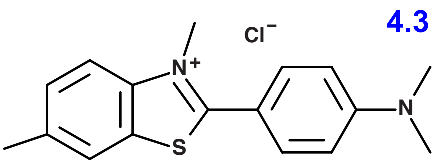

ThT (Thioflavin-T)

Supplementary Figure 3: Chemical structures of fluorophores tested in this study (continued)

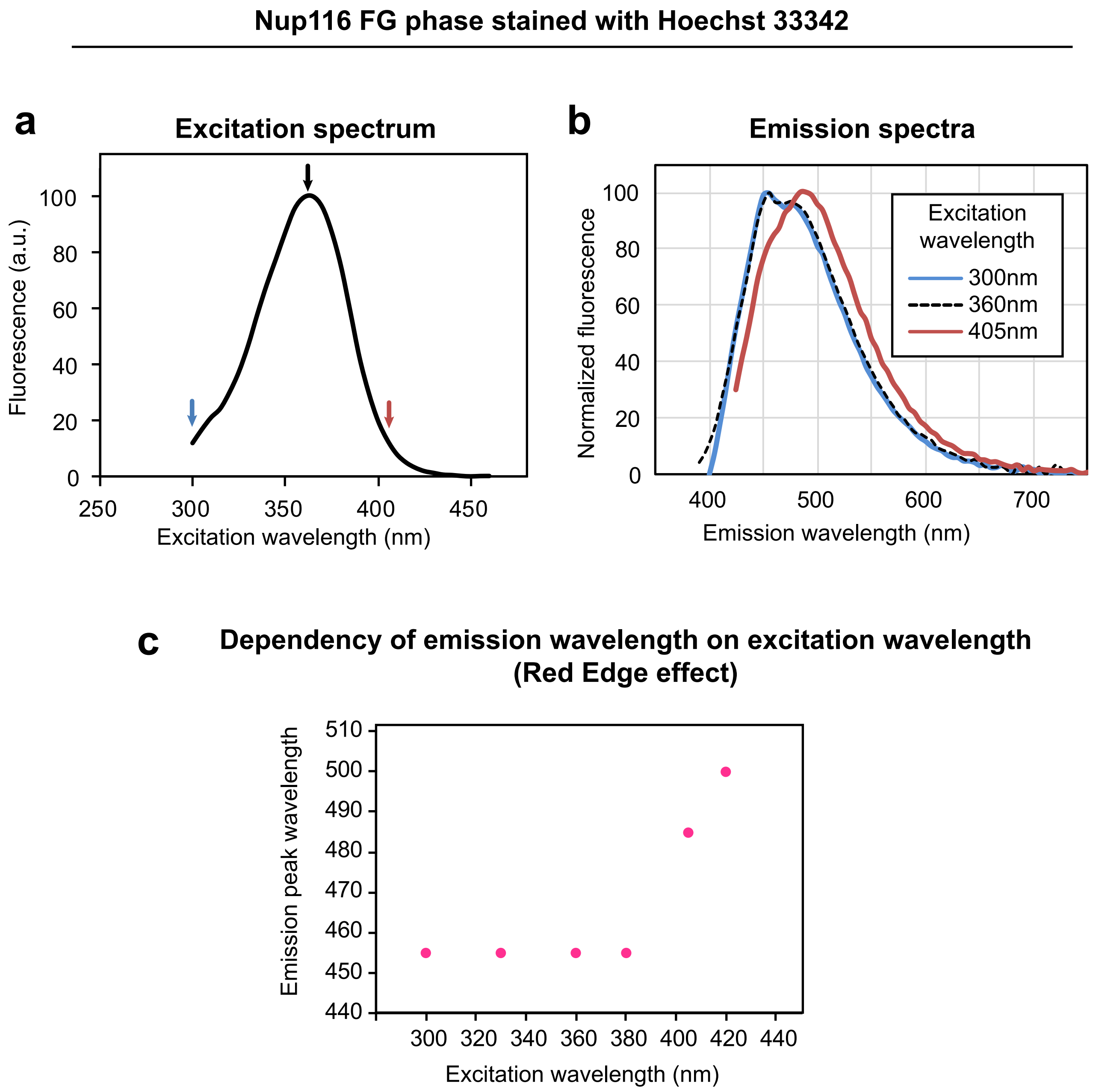

Supplementary Figure 4: Red edge effect observed in Hoechst 33342-stained FG phase
(a) Excitation spectrum of Hoechst 33342-stained Nup116 FG phase was recorded by a monochromator-based microplate reader between 300-460 nm with a 5 nm step size (detection wavelength: 475-485 nm). a.u.: arbitrary units.

(b) Emission spectra recorded at three distinct excitation wavelengths: 300nm corresponding to the 'blue edge' (see the blue arrow in (a)); 360nm corresponding to the peak (black arrow), and 405nm corresponding to the 'red edge' of the excitation spectrum (red arrow). Excitations at the blue edge and the peak showed overlapping emission spectra, while red edge excitation led to a red-shift of the emission spectrum and emission peak, known as the 'red edge effect'[3–6].

(c) The positions of the emission peaks were plotted against the excitation wavelengths, from the blue (300nm) to the red (420nm) edge of the excitation spectrum. A significant (up to 45nm) red-shift of the emission peak was observed between 380-420nm excitation (red edge region).

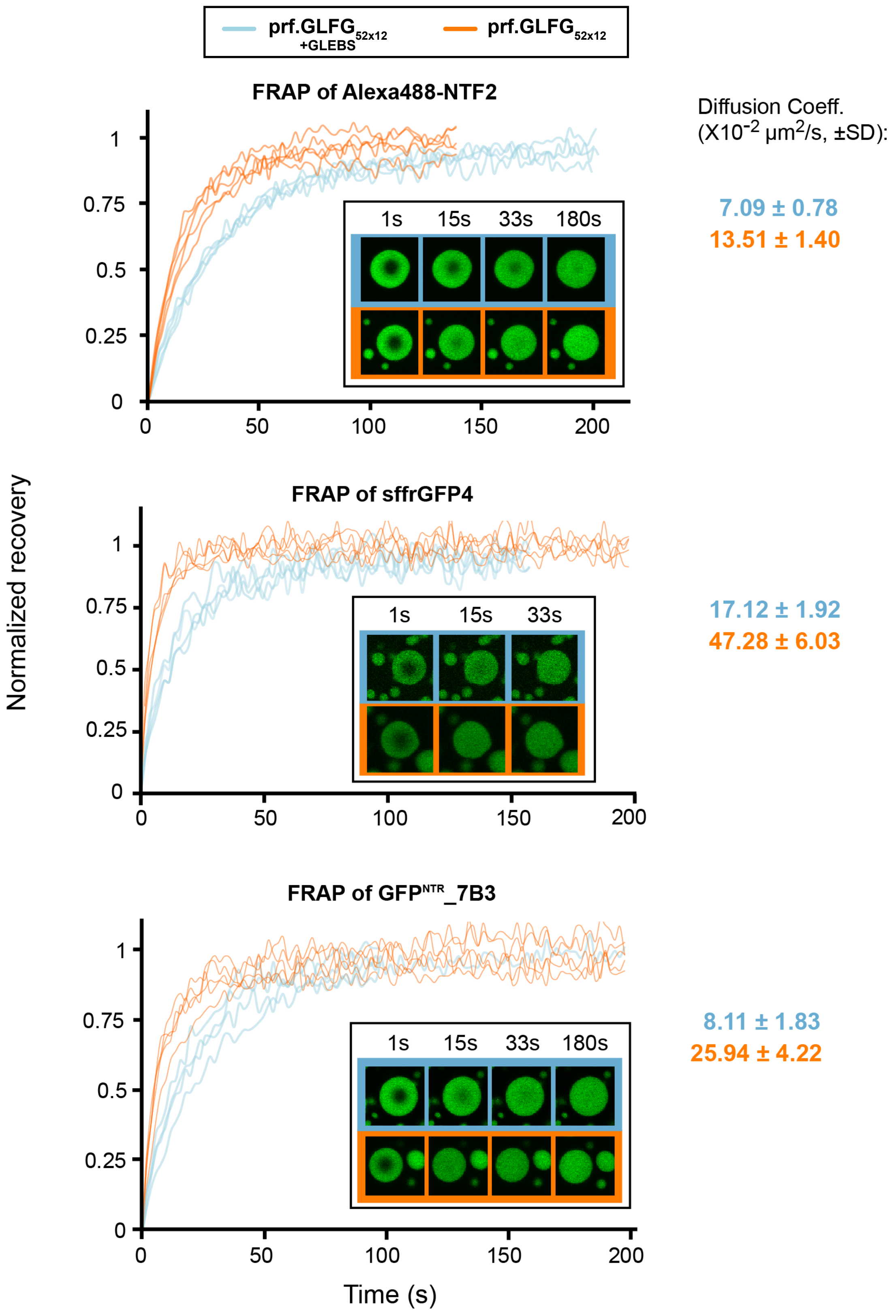

Supplementary Figure 5: Fluorescence recovery after photobleaching (FRAP) complete datasets
FRAP experiments with different fluorescent protein probes inside indicated FG phases (also described in Figure 7 and Methods). Experiments were typically repeated with multiple FG particles (n≥4). The recovery curves, average diffusion coefficients +/- standard deviations (SD) of the replicates and representative images (inset) are shown here. GFP^{NTR}_7B3 is a previously engineered GFP variant with NTR-like FG-philicity and rapid NPC-passage[7].

a

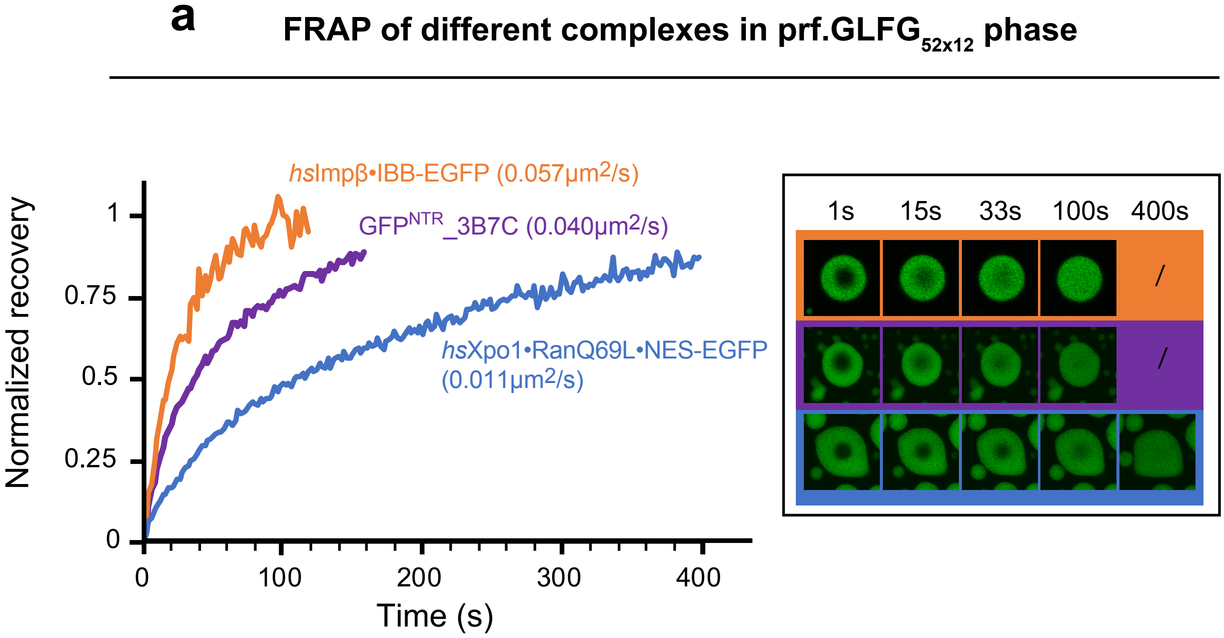

b

Diffusion coefficients ± SD (μm^2/s) in free solution				
NTF2	sffrGFP4	GFP^{NTR}_7B3	GFP^{NTR}_3B7C	prf.GLFG$_{52x12}$
70 ± 10	77 ± 13	63 ± 5	54 ± 3	36 ± 3

Supplementary Figure 6: Diffusivity of FG-philic species and complexes inside the prf.GLFG$_{52x12}$ phase

(a) FRAP experiments were performed inside the FG phase of (GLEBS-free) prf.GLFG$_{52x12}$, with different protein complexes (see the main text for details) as probes. 3B7C is a tetrameric GFP variant with high selectivity for FG phases and rapid NPC passage[7]. The numbers are diffusion coefficients estimated from the datasets.

(b) Diffusion coefficients of the indicated species in solution (assay buffer) at 21°C were measured by dynamic light scattering (DLS), using a DynaPro NanoStar instrument (Wyatt Technologies). The Dynamics 7.1.5 software was used for autocorrelation analysis. Average values and standard deviations (SD) were derived from 6 measurements. Measurements for prf.GLFG$_{52x12}$ were performed at a concentration below the critical concentration for phase separation.

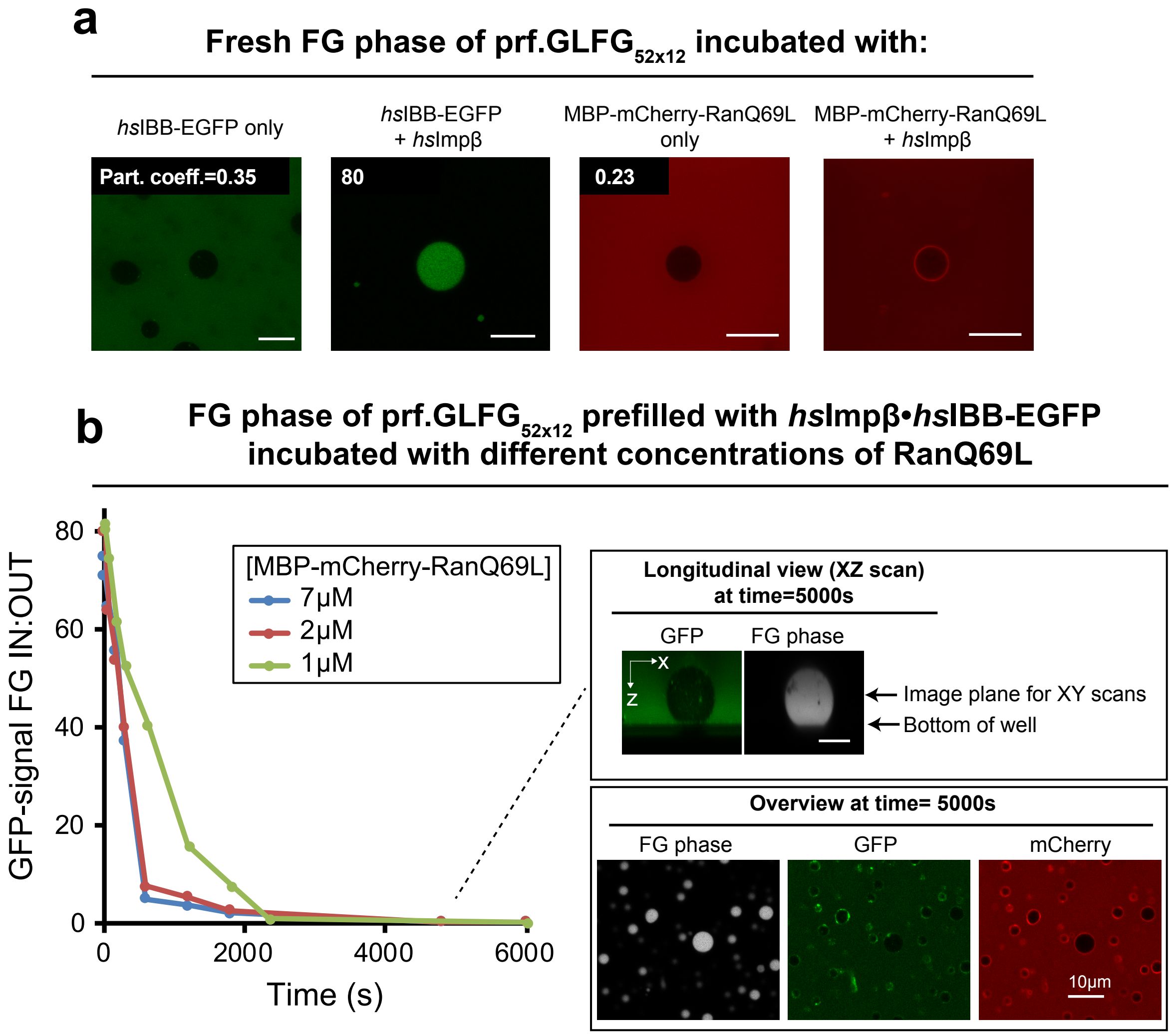

Supplementary Figure 7: Efflux of import cargo from the prf.GLFG$_{52x12}$ phase

(a) FG phase formed by prf.GLFG$_{52x12}$ was challenged with different proteins/ complexes. *hs*IBB-EGFP or MBP-mCherry-RanQ69L were excluded from the FG phase, unless the NTR, *hs*Impβ was present. The *hs*Impβ•MBP-mCherry-RanQ69L complex arrested at the rim of the FG particle, as expected from the FG-phobic effect of the MBP-mCherry group. Image brightness was adjusted individually. Scale bars: 10µm.

(b) Experiment described in Fig.8a-d was repeated with different initial concentrations of MBP-mCherry-RanQ69L. The quantification of GFP signal was based on particles of diameters ≈15µm. The results showed that RanGTP was no longer rate-limiting when used at ≥2µM concentrations. 7µM of MBP-mCherry-RanQ69L was used in Fig.8a-d. The FG phase was visualized by Alexa647-labeled FG domain molecules, as described in Fig.7c. At time=5000s, the cargo (IBB-EGFP) was almost completely excluded from the FG particles (right) and MBP-mCherry-RanQ69L had entered the rim of the particles, indicating that Impβ had bound and recruited the Ran fusion. Scale bars: 10µm.

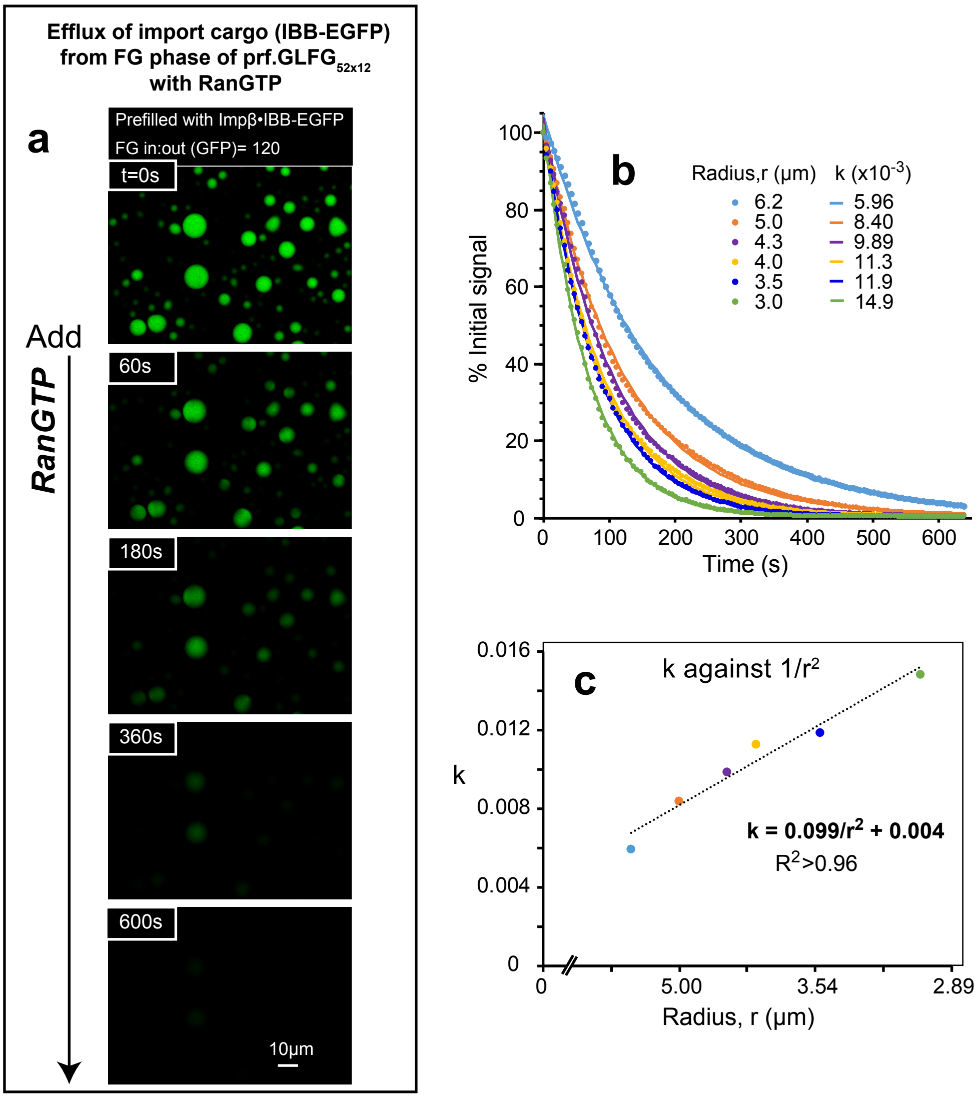

RanGTP: Ran protein pre-incubated with RanGEF and an ATP/GTP-regenerating system

Supplementary Figure 8: Efflux of import cargo from the prf.GLFG$_{52x12}$ phase driven by a wild-type RanGTP

Experiment described in Fig.8 was repeated with a (non-fused) wild-type Ran protein, which had been pre-incubated with RanGEF and an ATP/GTP-regenerating system for quantitative GTP-loading.

(a) The FG phase of prf.GLFG$_{52x12}$ was initially loaded with *hs*Impβ•*hs*IBB-EGFP complex. At time=0s, the wild-type Ran protein was added, which triggered the unloading of IBB-EGFP from Impβ and thus an efflux from the FG phase. Fluorescence signal of GFP was recorded over time.

(b) Time course of IBB-EGFP signal (normalized to % of the initial signal) inside FG particles with different radii. Solid lines: best-fits to a single exponential decay function: $f(t) = Ae^{-kt} + B$, where t is the time and k is the respective time constant. Source data are provided as a Source Data file.

(c) k obtained was plotted against $1/r^2$, where r is the FG particle's radius.

Note that the efflux was ~ two-fold faster than the efflux driven by MBP-mCherry-RanQ69L (Fig.8). This may be explained by faster diffusion of the non-fused Ran.

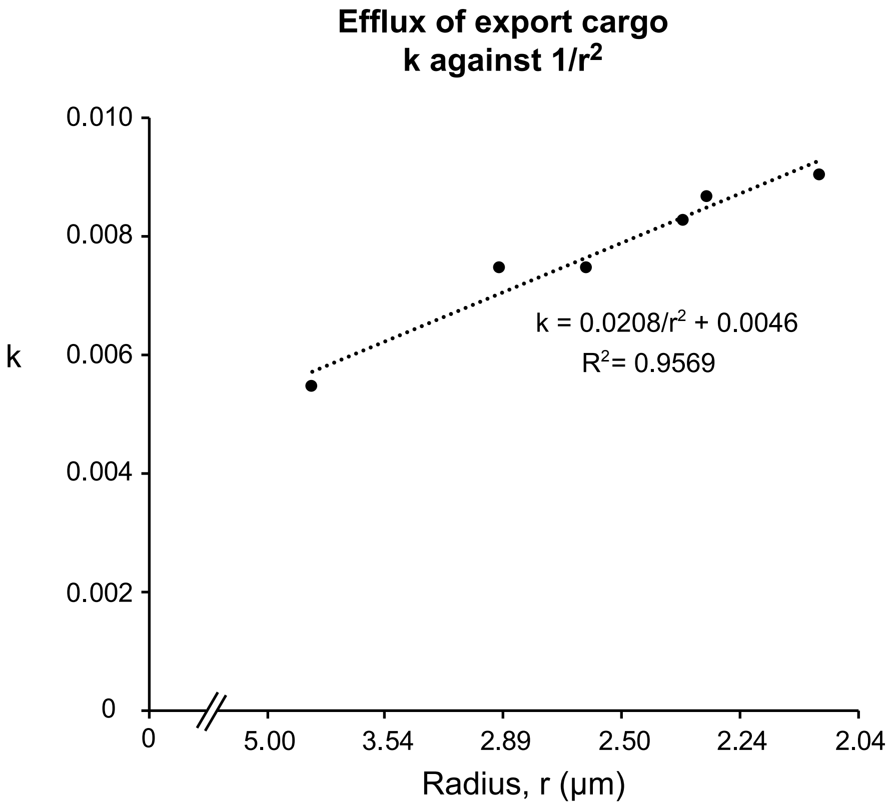

Supplementary Figure 9: Scaling of export cargo efflux rate with FG particle size
k obtained in the experiment described in Fig.9c,d was plotted against $1/r^2$, where r is the radius of the FG particle. Additional data points were added from the same image set.

	Mac98A FG domain	Variant T1	Variant T2	$GLFG_{52x12}$	Pro-free $prf.GLFG_{52x12}$	$prf.GLFG_{52x12}$ [+GLEBS]
Total amino acids	666	666	666	667	668	668
**GLFG motifs**	27	36	51	52	52	52
Other FG motifs	15	11	1	0	0	0
FG-like motifs	14	9	0	0	0	0
Isolated hydrophobic AA	3	3	0	0	0	0
Amino acid frequencies (%)						
A	10.6	10.6	10.6	10.6	8.3	8.3
C	0.0	0.0	0.0	0.0	0.0	0.0
D	0.0	0.0	0.0	0.0	0.0	0.0
E	0.0	0.0	0.0	0.0	0.0	0.0
F	7.6	8.2	8.4	8.3	8.3	8.3
G	34.1	34.1	34.1	34.2	33.3	33.3
H	0.0	0.0	0.0	0.0	0.0	0.0
I	0.6	0.2	0.0	0.0	0.0	0.0
K	0.3	0.5	0.5	0.5	0.0	0.0
L	6.9	7.4	8.2	8.3	8.3	8.3
M	1.4	0.8	0.2	0.0	0.0	0.0
N	10.5	10.5	10.5	10.4	8.3	8.3
P	4.8	4.8	4.8	4.8	0.0	8.3
Q	8.4	8.4	8.4	8.3	8.3	8.3
R	0.2	0.0	0.0	0.0	0.0	0.0
S	1.4	1.4	1.4	1.4	8.3	0.0
T	13.0	13.0	13.0	13.0	16.7	16.7
V	0.2	0.2	0.0	0.0	0.0	0.0
W	0.0	0.0	0.0	0.0	0.0	0.0
Y	0.0	0.0	0.0	0.0	0.0	0.0
**Sum of F+I+L+M+V**	**16.7**	**16.8**	**16.8**	**16.6**	**16.6**	**16.6**

Supplementary Table 1: Number of FG- or FG-like motifs and amino acid frequencies (%) of the wild-type MacNup98A (Mac98A) FG domain and derived variants. Note: Composition of the GLEBS domain was ignored from the calculations. FG-like motifs include: Fx, LG, MG and IG dipeptides, where x is any amino acid.

FG domain	Fluorescence enhancement of Hoechst 33342 (fold)	Conc. FG domain		Conc. FG dipeptides (µM)	Conc. Phe (µM)
		mg/ml	µM		
Nup116	160	2.7	34	1620	2100
Mac98A	64	2.7	41	1720	2000
$GLFG_{52x12}$	83	2.7	41	2130	2210
Nsp1 274-601 (Non-cohesive)	2.1	2.7	71	1210	2410
**Plasmid**	670	n.a.	n.a.	n.a.	n.a.

Supplementary Table 2: Fluorescence enhancements of Hoechst 33342 upon binding to FG domains/ DNA. In the bulk measurements of fluorescence (Fig.4b), the samples were standardized by µg per µl: 400µg of each sample was diluted in 150µl volume, containing 20µM of Hoechst 33342 (dye). Fluorescence enhancement is the difference of fluorescence signal measured with and without FG domain (or DNA) divided by the fluorescence without FG domain (or DNA) (see below). Molar masses, and thus molar concentrations of different samples were different. Note: the GLEBS domain (in Nup116, Mac98A and $GLFG_{52x12}$) was included in the calculations of Phe concentration. (n.a.= not applicable)

$$Fluorescence\ enhancement = \frac{[Signal(Dye + FG\ domain)] - [Signal\ (Dye)]}{Signal(Dye)}$$

(all signals have been corrected by subtracting the instrumental background)

Probe	Molecular weight (kDa)	Phase	Diffusion coefficient (μm²/s)	Distance diffused in 10ms (nm)
**Alexa488-NTF2**	30	Solution	70	1200
		prf.GLFG$_{52x12}$ [+GLEBS]	0.07	38
		prf.GLFG$_{52x12}$	0.13	51
**sffrGFP4**	27	Solution	77	1200
		prf.GLFG$_{52x12}$ [+GLEBS]	0.17	59
		prf.GLFG$_{52x12}$	0.47	97
**GFP^{NTR}_7B3**	27	Solution	63	1100
		prf.GLFG$_{52x12}$ [+GLEBS]	0.08	40
		prf.GLFG$_{52x12}$	0.26	72
**GFP^{NTR}_3B7C**	110	prf.GLFG$_{52x12}$	0.04	28
***hs*Impβ• IBB-EGFP**	130	prf.GLFG$_{52x12}$	0.06	34
***hs*Xpo1•RanQ69L• NES-EGFP**	170	prf.GLFG$_{52x12}$	0.01	15

Supplementary Table 3: Parameters scaled down to nuclear pore size. Diffusion coefficients obtained in this study (Fig.7 and Supplementary Fig.5,6) are correlated to distances (root mean square displacement *r.m.s.d.*) travelled within 10 milliseconds: the typical timescale of a nuclear translocation event[8], by Fick's laws of diffusion and a 1D random walk model:

$$\bar{d} = \sqrt{2Dt} \quad \text{or written as:}$$

$$t = \frac{(\bar{d})^2}{2D}$$

where $\bar{d}$ is the root mean square displacement from the starting point. *D* is the measured diffusion coefficient and *t* is the time.

Protein name	Plasmid	Encoding for	Used in figures	Reference
*Tt*MacNup98A (Mac98A) FG domain	pHBS418	His_{18}-*Tt*MacNup98A$_{1-666}$-Cys	1,2,3,4,6,7,S1, S2	9
Variant T1	pSNG025	His_{18}-Variant T1 -Cys	1	this study
Variant T2	pSNG026	His_{18}-Variant T2 -Cys	1	this study
GLFG$_{52x12}$	pSNG036	His_{18}- GLFG$_{52x12}$-Cys	1,4	this study
*sc*Nup116 FG domain	pHBS698	His_{18}-*sc*Nup116$_{1-736}$-Cys	2,3,4,S1,S2,S4	9
*sc*Nsp1$_{274-601}$ FG	pSF654	His_{10}-TEV-*sc*Nsp1$_{274-601}$ -Cys	4	10
Pro-free_prf.GLFG$_{52x12}$	pSNG037	His_{18}- Pro-free_prf.GLFG$_{52x12}$-Cys	2,4,S1	this study
prf.GLFG$_{52x12}$+GLEBS	pSNG038	His_{18}- prf.GLFG$_{52x12}$[+GLEBS] -Cys	1,2,4,6,7,S1,S5	this study
prf.GLFG$_{52x12}$	pSNG053	His_{18}- prf.GLFG$_{52x12}$-Cys	6,7,8,9,S5-9	this study
Mac98A ΔGLEBS	pSNG034	His_{18}-*Tt*MacNup98A$_{1-666\ \Delta 336-365}$-Cys	6	this study
Proline-free Mac98A	pSNG115	His_{18}-*Tt*MacNup98AΔPro-Cys	S2	this study
NTF2	pDG2121	rat NTF2	1,2,6,7,S2,S5, S6	7
*sc*Importin β (*sc*Impβ)*	pMR676	His_{14}-*bd*SUMO-*sc*Kap95p	6	9
*hs*Importin β (*hs*Impβ)*	pDG2305	His_{14}-MBP-*bd*SUMO-*hs*Imp_beta	5,6,8,S6,S7,S8	7
*sc*IBB-EGFP*	pSF807	His_{14}-TEV-*sc*Srp1p$_{2-63}$-mEGFP	6	9
*hs*IBB-EGFP*	pDG2895	His_{14}-*bd*SUMO-IBB-EGFP	6,8,S6,S7,S8	7
*hs*IBB-sinGFP4a*	pDG2894	His_{14}-*bd*SUMO-IBB-sinGFP4a	5	7
*sc*IBB-MBP-EGFP-*hs*M9*	pHBS704	His_{14}-TEV-*sc*Srp1p$_{2-63}$-MBP-mEGFP-hnRNPA1$_{268-306}$	6	9
efGFP_8Q*	pDG2936	His_{14}-*bd*SUMO-efGFP_8Q	3,4	7
efGFP_8Q-Cys*	pDG3233	His_{14}-*bd*SUMO-efGFP_8Q-Cys	3	this study
sffrGFP4*	pDG2805	His_{14}-*bd*SUMO-sffrGFP4	6,S5,S6	7
sffrGFP7*	pDG2715	His_{14}-*bd*SUMO-sffrGFP7	4	7
sffrGFP4 25xR→K*	pSF2885	His_{14}-*bd*SUMO-sffrGFP4 complete R-K mutant	6	7
GFP^{NTR}_7B3*	pDG2798	His_{14}-*bd*SUMO-GFP^{NTR}7B3	S5,S6	7
GFP^{NTR}_3B7C*	pDG2779	His_{14}-*bd*SUMO-GFP^{NTR}3B7C	6,S6	7
*hs*Xpo1/*hs*CRM1*	pTG-A42	His_{10}-ZZ-TEV-*hs*CRM1	6,9,S6,S9	11
NES-EGFP*	pTG-A450	His_{14}-TEV-PKI01-mEGFP-Cys	6,9,S6,S9	this study
mCherry*	pSF779	His_{14}-TEV-mCherry-Cys	1,2,4,5,6,S2	9
RanQ69L$_{1-180}$	pTG-A418	His_{10}-ZZ-TEV-*hs*RanQ69L $_{1-180}$	6,S6	11
MBP-mCherry-RanQ69L*	pSNG077	His_{14}-*bd*SUMO-MBP-mCherry-*hs*RanQ69L $_{1-180}$	8,S7	this study
*hs*RanGTP/GDP*	pDG2960	His_{14}-ZZ-*sc*SUMO-*hs*Ran	6,9,S8,S9	this study
RanGEF	pSF815	His_{14}-TEV-*sc*Prp20	6,9,S8,S9	this study
RanGAP	pDG2130	zz-Rna1p-His_{6}	6,9,S9	12

Supplementary Table 4: Proteins and corresponding bacterial expression constructs used in this study. Plasmid numbers are unique identifiers. * indicates that a His-tag-cleaved (by SUMO/ TEV) version was used.

Supplementary Note 1:
Complete amino acid sequences of engineered FG domain variants and reference wild-type FG domains

*Coloured in red: GLEBS domain

Wild-type *Tetrahymena thermophila* Macronuclear Nup98A FG domain (MacNup98A/Mac98A FG domain)
Plasmid: pHBS418

MFGNTGGGGLFGNTQTQQTGGGLFGQPQQTQFGQTGATGGGLFGGATNTFGGGGGGLFGGNNNQQTNPTAGGGI
FGQGTTGLGGAPAQTGGGLFGAPQNNQGGGLFGGGTTTGGGMFGNQANTQTGGGGLFGGPSQPTTQPPAFSLNNP
TTGGGGLFGQPANTMGGNNGGLFGGQTNSFGANNNMLGNNNRPQGAGIFGGATTTAPTGNTGMFGGIGANNGGGG
LFGMNNTNTNPTGGFGATNPTAGGGGLFGGGATTTGGGGLFGGGNTQGGGLLGTANTTAGGLLGGGFNMNNNTGG
ILGQTNNQFGLGSFGTNNNAAAAPFQPKASANGVLTKPNEKNLCYAISNGTDFCIFELALTQRKLVKAGQLKPGA
QQAGGMFGQPAQGGNGLFGGGGAATTTPFGGAQNGNLFGGQNTQAQGGGLFGAPVNNAATGAGGGLFGAKPAATT
TGGGLFGQMPAQTGGFLGNTATQPAGGGLFGGATTTQAPGGGGGGGLFGGNTTAATTGGGLFGGNTQTGGATGGL
FGGQQPNNQGGLFLNTGNANNANTGGGLFGGATTTPATGGGLFGGSTNTQPGLATGGGLFGNNQGASQPAAQGGL
FGGAAPQQNSLFGGATAGGQTGGLFGGATGATQQQGGGLFGQTASNPTQGGGLFGAANPGLGGAAA

Variant T1
Plasmid: pSNG025

MFGNTGGGGLFGNTQTQQTGGGLFGQPQQTQFGQTGATGGGLFGGATNTFGGGGGGLFGGNNNQQTNPTAGGGL
FGQGTTGLGGAPAQTGGGLFGAPQNNQGGGLFGGGTTTGGGLFGNQANTQTGGGGLFGGPSQPTTQPPALFSNNP
TTGGGGLFGQPANTMGGNNGGLFGGQTNSFGANNNLFGNNNKPQGAGLFGGATTTAPTGNTGLFGGIGANNGGGG
LFGMNNTNTNPTGGFGATNPTAGGGGLFGGGATTTGGGGLFGGGNTQGGGLFGTANTTAGGLFGGGFNMNNNTGG
LFGQTNNQLFGGSFGTNNNAAAAPFQKPASANGVLTKPNEKNLCYAISNGTDFCIFELALTQRKLVKAGQLKPGA
QQAGGLFGQPAQGGNGLFGGGGAATTTPFGGAQNGNLFGGQNTQAQGGGLFGAPVNNAATGAGGGLFGAKPAATT
TGGGLFGQMPAQTGGLFGNTATQPAGGGLFGGATTTQAPGGGGGGGLFGGNTTAATTGGGLFGGNTQTGGATGGL
FGGQQPNNQGGLFLNTGNANNANTGGGLFGGATTTPATGGGLFGGSTNTQPGLATGGGLFGNNQGASQPAAQGGL
FGGAAPQQNSLFGGATAGGQTGGLFGGATGATQQQGGGLFGQTASNPTQGGGLFGAANPGLGGAAA

Variant T2
Plasmid: pSNG026

MFGNTGGGGLFGNTQTQQTGGGLFGQPQQTQGGGLFGQTGATGGGLFGGATNTGGLFGGGGGGGLFGGNNNQQTN
PTAGGLFGQGTTAPAQTGGGLFGAPQNNQGGGLFGGGTTTGGGLFGNQANTQTGGGGLFGGPSQPTTQPPAGLFG
SNNPTTGGGLFGQPANTNNGGLFGGQTNSGLFGANNNGLFGNNNKPQAGLFGGATTTAPTGNTGLFGGANNGGGG
LFGNNTNTNPTGGLFGATNPTAGGGGLFGGGATTTGGGGLFGGGNTQGGGLFGTANTTAGGLFGGGNGLFGNNNT
GGLFGQTNNQGLFGGTNNNAAAAPSGLFGQKPASANGVLTKPNEKNLCYAISNGTDFCIFELALTQRKLVKAGQL
KPGAQQAGGLFGQPAQNGGLFGGGGAATTTPGLFGGAQNNGLFGGQNTQAQGGGLFGAPNNAATGLFGAGGGLFG
AKPAATTTGGGLFGQPAQTGGLFGNTATQPAGGGLFGGATTTQAPGGGLFGGNTTAATTGGGLFGGNTQTGGATG
GLFGGQQPNNQGGLFGNTNANNANTGGGLFGGATTTPATGGGLFGGSTNTQPGATGGGLFGNNQGASQPAAQGGL
FGGAAPQQNSGLFGGATAGQTGGLFGGATATQQQGGGLFGQTASNPTQGGGLFGAANPGLFGGAAA

$GLFG_{52x12}$
Plasmid: pSNG036

GLFGNTGGAPAGGLFGNTQTQQGGGLFGQPQQTQGGGLFGQTGATTGGGLFGGATNTAPGGLFGGGGGNPTGGLF
GGNNNQQTGGLFGQGTTQTGGGLFGAPQNNQGGGLFGGGTTTTGGGLFGANTQTGGGGLFGGPSQPTTAGLFGSN
NPTTGGGLFGQPANTNNGGLFGGQTNNQASGLFGANNQPPTNGLFGNNNKPQTAGLFGGATTTGNTGLFGGANNT
GGGGLFGNNTNNPTGGLFGATNPAGGGGLFGGGATTGGGGLFGGGNTQTGGGLFGTANTTTAGGLFGGGNTQPQN
GLFGNNNTPATGGLFGQTNNAAPQGLFGGTNNNAASGLFGQKPASANGVLTKPNEKNLCYAISNGTDFCIFELAL
TQRKLVKAGQLKPGAQAGGLFGQPAQNTQGGLFGGGGAATTPGLFGGAQNNTTGGLFGGQNTQAGGGLFGAPNNA
AATGLFGAGNANTQGGLFGAKPAATGGGLFGQPAQTQAGGLFGNTAQPAGGGLFGGATTTPGGGLFGGNTAATGG
GLFGGNTQGATGGLFGGQQPNNQGGLFGNTNANTGGGLFGGATTTTGGGLFGGSTGATGGGLFGGASQPAAGGLF
GGAAPQQNSGLFGGATAGQTGGLFGGATQQQGGGLFGQTASNPGGGLFGAANATTQPGLFGGNNQAA

Pro-free_ prf.GLFG$_{52x12}$
Plasmid: pSNG037
GGLFGGATNSQTGGLFGGATNSQTGGLFGGATNSQTGGLFGGATNSQTGGLFGGATNSQTGGLFGGATNSQTGGL
FGGATNSQTGGLFGGATNSQTGGLFGGATNSQTGGLFGGATNSQTGGLFGGATNSQTGGLFGGATNSQTGGLFGG
ATNSQTGGLFGGATNSQTGGLFGGATNSQTGGLFGGATNSQTGGLFGGATNSQTGGLFGGATNSQTGGLFGGATN
SQTGGLFGGATNSQTGGLFGGATNSQTGGLFGGATNSQTGGLFGGATNSQTGGLFGGATNSQTGGLFGGATNSQT
GGLFGGATNSQTGGLFGGATNSQTGGLFGGATNSQTGGLFGGATNSQTGVLTKPNEKNLCYAISNGTDFCIFELA
LTQRKLVKAGQLKPGAQGGLFGGATNSQTGGLFGGATNSQTGGLFGGATNSQTGGLFGGATNSQTGGLFGGATNS
QTGGLFGGATNSQTGGLFGGATNSQTGGLFGGATNSQTGGLFGGATNSQTGGLFGGATNSQTGGLFGGATNSQTG
GLFGGATNSQTGGLFGGATNSQTGGLFGGATNSQTGGLFGGATNSQTGGLFGGATNSQTGGLFGGATNSQTGGLF
GGATNSQTGGLFGGATNSQTGGLFGGATNSQTGGLFGGATNSQTGGLFGGATNSQTGGLFGGATNSQT

prf.GLFG$_{52x12}$[+GLEBS]
Plasmid: pSNG038
GGLFGGNTQPATGGLFGGNTQPATGGLFGGNTQPATGGLFGGNTQPATGGLFGGNTQPATGGLFGGNTQPATGGL
FGGNTQPATGGLFGGNTQPATGGLFGGNTQPATGGLFGGNTQPATGGLFGGNTQPATGGLFGGNTQPATGGLFGG
NTQPATGGLFGGNTQPATGGLFGGNTQPATGGLFGGNTQPATGGLFGGNTQPATGGLFGGNTQPATGGLFGGNTQ
PATGGLFGGNTQPATGGLFGGNTQPATGGLFGGNTQPATGGLFGGNTQPATGGLFGGNTQPATGGLFGGNTQPAT
GGLFGGNTQPATGGLFGGNTQPATGGLFGGNTQPATGGLFGGNTQPATGVLTKPNEKNLCYAISNGTDFCIFELA
LTQRKLVKAGQLKPGAQGGLFGGNTQPATGGLFGGNTQPATGGLFGGNTQPATGGLFGGNTQPATGGLFGGNTQP
ATGGLFGGNTQPATGGLFGGNTQPATGGLFGGNTQPATGGLFGGNTQPATGGLFGGNTQPATGGLFGGNTQPATG
GLFGGNTQPATGGLFGGNTQPATGGLFGGNTQPATGGLFGGNTQPATGGLFGGNTQPATGGLFGGNTQPATGGLF
GGNTQPATGGLFGGNTQPATGGLFGGNTQPATGGLFGGNTQPATGGLFGGNTQPATGGLFGGNTQPAT

prf.GLFG$_{52x12}$
Plasmid: pSNG053
GGLFGGNTQPATGGLFGGNTQPATGGLFGGNTQPATGGLFGGNTQPATGGLFGGNTQPATGGLFGGNTQPATGGL
FGGNTQPATGGLFGGNTQPATGGLFGGNTQPATGGLFGGNTQPATGGLFGGNTQPATGGLFGGNTQPATGGLFGG
NTQPATGGLFGGNTQPATGGLFGGNTQPATGGLFGGNTQPATGGLFGGNTQPATGGLFGGNTQPATGGLFGGNTQ
PATGGLFGGNTQPATGGLFGGNTQPATGGLFGGNTQPATGGLFGGNTQPATGGLFGGNTQPATGGLFGGNTQPAT
GGLFGGNTQPATGGLFGGNTQPATGGLFGGNTQPATGGLFGGNTQPATGGLFGGNTQPATGGLFGGNTQPATGGL
FGGNTQPATGGLFGGNTQPATGGLFGGNTQPATGGLFGGNTQPATGGLFGGNTQPATGGLFGGNTQPATGGLFGG
NTQPATGGLFGGNTQPATGGLFGGNTQPATGGLFGGNTQPATGGLFGGNTQPATGGLFGGNTQPATGGLFGGNTQ
PATGGLFGGNTQPATGGLFGGNTQPATGGLFGGNTQPATGGLFGGNTQPATGGLFGGNTQPATGGLFGGNTQPAT
GGLFGGNTQPATGGLFGGNTQPAT

Wild-type *Saccharomyces cerevisiae* Nup116 FG domain
plasmid: pHBS698
MFGVSRGAFPSATTQPFGSTGSTFGGQQQQQQPVANTSAFGLSQQTNTTQAPAFGNFGNQTSNSPFGMSGSTTAN
GTPFGQSQLTNNNASGSIFGGMGNNTALSAGSASVVPNSTAGTSIKPFTTFEEKDPTTGVINVFQSITCMPEYRN
FSFEELRFQDYQAGRKFGTSQNGTGTTFNNPQGTTNTGFGIMGNNNSTTSATTGGLFGQKPATGMFGTGTGSGGG
FGSGATNSTGLFGSSTNLSGNSAFGANKPATSGGLFGNTTNNPTNGTNNTGLFGQQNSNTNGGLFGQQQNSFGAN
NVSNGGAFGQVNRGAFPQQQTQQGSGGIFGQSNANANGGAFGQQQGTGALFGAKPASGGLFGQSAGSKAFGMNTN
PTGTTGGLFGQTNQQQSGGGLFGQQQNSNAGGLFGQNNQSQNQSGLFGQQNSSNAFGQPQQQGGLFGSKPAGGLF
GQQQGASTFASGNAQNNSIFGQNNQQQQSTGGLFGQQNNQSQSQPGGLFGQTNQNNNQPFGQNGLQQPQQNNSLF
GAKPTGFGNTSLFSNSTTNQSNGISGNNLQQQSGGLFQNKQQPASGGLFGSKPSNTVGGGLFGNNQVANQNNPAS
TSGGLFGSKPATGSLFGGTNSTAPNASSGGIFGSNNASNTAATTNSTGLFGNKPVGAGASTSAGGLFGNNNNSSL
NNSNGSTGLFGSNNTSQSTNAGGLFQNNTSTNTSGGGLFSQPSQSMAQSQNALQQQQQQR

Wild-type *Saccharomyces cerevisiae* Nsp1 residues 274-601 ('non-cohesive Nsp1 FG fragment')
Plasmid: pSF654
DNKTTNTTPSFSFGAKSDENKAGATSKPAFSFGAKPEEKKDDNSSKPAFSFGAKSNEDKQDGTAKPAFSFGAKPA
EKNNNETSKPAFSFGAKSDEKKDGDASKPAFSFGAKPDENKASATSKPAFSFGAKPEEKKDDNSSKPAFSFGAKS
NEDKQDGTAKPAFSFGAKPAEKNNNETSKPAFSFGAKSDEKKDGDASKPAFSFGAKSDEKKDSDSSKPAFSFGTK
SNEKKDSGSSKPAFSFGAKPDEKKNDEVSKPAFSFGAKANEKKESDESKSAFSFGSKPTGKEEGDAKAAISFGA
KPEEQKSSDTSKPAFTFGAQKDNEKKTE

Mac98A ΔGLEBS
Plasmid: pSNG034

MFGNTGGGGLFGNTQTQQTGGGLFGQPQQTQFGQTGATGGGLFGGATNTFGGGGGGLFGGNNNQQTNPTAGGGI
FGQGTTGLGGAPAQTGGGLFGAPQNNQGGGLFGGGTTTGGGMFGNQANTQTGGGGLFGGPSQPTTQPPAFSLNNP
TTGGGGLFGQPANTMGGNNGGLFGGQTNSFGANNNMLGNNNRPQGAGIFGGATTTAPTGNTGMFGGIGANNGGGG
LFGMNNTNTNPTGGFGATNPTAGGGGLFGGGATTTGGGGLFGGGNTQGGGLLGTANTTAGGLLGGGFNMNNNTGG
ILGQTNNQFGLGSFGTNNNAAAAPFQPKASANGVLVKAGQLKPGAQQAGGMFGQPAQGGNGLFGGGGAATTTPFG
GAQNGNLFGGQNTQAQGGGLFGAPVNNAATGAGGGLFGAKPAATTTGGGLFGQMPAQTGGFLGNTATQPAGGGLF
GGATTTQAPGGGGGGGLFGGNTTAATTGGGLFGGNTQTGGATGGLFGGQQPNNQGGLFLNTGNANNANTGGGLFG
GATTTPATGGGLFGGSTNTQPGLATGGGLFGNNQGASQPAAQGGLFGGAAPQQNSLFGGATAGGQTGGLFGGATG
ATQQQGGGLFGQTASNPTQGGGLFGAANPGLGGAAA

Proline-free Mac98A
Plasmid: pSNG115

MFGNTGGGGLFGNTQTQQTGGGLFGQAQQTQFGQTGATGGGLFGGATNTFGGGGGGLFGGNNNQQTNNTAGGGI
FGQGTTGLGGAQAQTGGGLFGAGQNNQGGGLFGGGTTTGGGMFGNQANTQTGGGGLFGGASQGTTQTAAFSLNNT
TTGGGGLFGQNANTMGGNNGGLFGGQTNSFGANNNMLGNNNRGQGAGIFGGATTTAATGNTGMFGGIGANNGGGG
LFGMNNTNTNTTGGFGATNGTAGGGGLFGGGATTTGGGGLFGGGNTQGGGLLGTANTTAGGLLGGGFNMNNNTGG
ILGQTNNQFGLGSFGTNNNAAAAGFQNKASANGVLTKPNEKNLCYAISNGTDFCIFELALTQRKLVKAGQLKPGA
QQAGGMFGQMAQGGNGLFGGGGAATTTTFGGAQNGNLFGGQNTQAQGGGLFGATVNNAATGAGGGLFGAKGAATT
TGGGLFGQMQAQTGGFLGNTATQNAGGGLFGGATTTQAGGGGGGGLFGGNTTAATTGGGLFGGNTQTGGATGGL
FGGQQTNNQGGLFLNTGNANNANTGGGLFGGATTTQATGGGLFGGSTNTQAGLATGGGLFGNNQGASQGAAQGGL
FGGAANQQNSLFGGATAGGQTGGLFGGATGATQQQGGGLFGQTASNATQGGGLFGAANQGLGGAAA

Supplementary References

1. Serio, T. R. et al. Nucleated conformational conversion and the replication of conformational information by a prion determinant. *Science* **289**, 1317-1321 (2000).
2. Patel, A. et al. A Liquid-to-Solid Phase Transition of the ALS Protein FUS Accelerated by Disease Mutation. *Cell* **162**, 1066-1077 (2015).
3. Chattopadhyay, A. & Haldar, S. Dynamic insight into protein structure utilizing red edge excitation shift. *Acc Chem Res* **47**, 12-19 (2014).
4. Demchenko, A. P. The red-edge effects: 30 years of exploration. *Luminescence* **17**, 19-42 (2002).
5. Demchenko, A. P. Site-selective Red-Edge effects. *Methods Enzymol* **450**, 59-78 (2008).
6. Mukherjee, S. & Chattopadhyay, A. Wavelength-selective fluorescence as a novel tool to study organization and dynamics in complex biological systems. *J Fluoresc* **5**, 237-246 (1995).
7. Frey, S. et al. Surface properties determining passage rates of proteins through nuclear pores. *Cell* **174**, 202-217.e9 (2018).
8. Tu, L. C. & Musser, S. M. Single molecule studies of nucleocytoplasmic transport. *Biochim Biophys Acta* **1813**, 1607-1618 (2011).
9. Schmidt, H. B. & Görlich, D. Nup98 FG domains from diverse species spontaneously phase-separate into particles with nuclear pore-like permselectivity. *Elife* **4**, e04251 (2015).
10. Ader, C. et al. Amyloid-like interactions within nucleoporin FG hydrogels. *Proc Natl Acad Sci USA* **107**, 6281-6285 (2010).
11. Güttler, T. et al. NES consensus redefined by structures of PKI-type and Rev-type nuclear export signals bound to CRM1. *Nat Struct Mol Biol* **17**, 1367-1376 (2010).
12. Görlich, D., Pante, N., Kutay, U., Aebi, U. & Bischoff, F. R. Identification of different roles for RanGDP and RanGTP in nuclear protein import. *EMBO J* **15**, 5584-5594 (1996).
